# FCGR3A and FCGR2A Genotypes Differentially Impact Allograft Rejection and Patients' Survival After Lung Transplant

**DOI:** 10.3389/fimmu.2019.01208

**Published:** 2019-06-12

**Authors:** Pascale Paul, Pascal Pedini, Luc Lyonnet, Julie Di Cristofaro, Anderson Loundou, Mathieu Pelardy, Agnes Basire, Françoise Dignat-George, Jacques Chiaroni, Pascal Thomas, Martine Reynaud-Gaubert, Christophe Picard

**Affiliations:** ^1^Department of Hematology, Hopital de la Conception, INSERM CIC-1409, Assistance Publique-Hôpitaux Marseille (AP-HM), Marseille, France; ^2^INSERM 1263, INRA, C2VN, Aix-Marseille Université (AMU), INSERM, Marseille, France; ^3^Établissement Français du Sang PACA-Corse 13005, Marseille, France; ^4^“Biologie des Groupes Sanguins”, UMR 7268 ADÉS Aix-Marseille Université/EFS/CNRS, Marseille, France; ^5^Département de santé Publique - EA 3279, Assistance Publique-Hôpitaux Marseille (AP-HM), Aix-Marseille Université, Marseille, France; ^6^Service de Chirurgie Thoracique et Transplantation Pulmonaire, CHU Nord Assistance Publique-Hôpitaux Marseille (AP-HM), Aix-Marseille Université, Marseille, France; ^7^Service de Pneumologie et Transplantation Pulmonaire, CHU Nord Assistance Publique-Hôpitaux Marseille (AP-HM) - IHU Méditerranée Infection Aix-Marseille-Université, Marseille, France

**Keywords:** Fc-gamma receptors, natural killer cells, lung transplantation, chronic lung allograft dysfunction, HLA antibodies, allograft rejection

## Abstract

Fc gamma receptors (FcγRs) play a major role in the regulation of humoral immune responses. Single-nucleotide polymorphisms (SNPs) of *FCGR2A* and *FCGR3A* can impact the expression level, IgG affinity and function of the CD32 and CD16 FcγRs in response to their engagement by the Fc fragment of IgG. The CD16 isoform encoded by *FCGR3A* [158V/V] controls the intensity of antibody-dependent cytotoxic alloimmune responses of natural killer cells (NK) and has been identified as a susceptibility marker predisposing patients to cardiac allograft vasculopathy after heart transplant. This study aimed to investigate whether *FCGR2A* and *FCGR3A* polymorphisms can also be associated with the clinical outcome of lung transplant recipients (LTRs). The SNPs of *FCGR2A* ([131R/H], rs1801274) and *FCGR3A* ([158V/F], rs396991) were identified in 158 LTRs and 184 Controls (CTL). The corresponding distribution of genotypic and allelic combinations was analyzed for potential links with the development of circulating donor-specific anti-HLA alloantibodies (DSA) detected at months 1 and 3 after lung transplant (LTx), the occurrence of acute rejection (AR) and chronic lung allograft dysfunction (CLAD), and the overall survival of LTRs. The *FCGR3A* [158V/V] genotype was identified as an independent susceptibility factor associated with higher rates of AR during the first trimester after LTx (HR 4.8, *p* < 0.0001, 95% CI 2.37–9.61), but it could not be associated with the level of CD16- mediated NK cell activation in response to the LTR's DSA, whatever the MFI intensity and C1q binding profiles of the DSA evaluated. The *FCGR2A* [131R/R] genotype was associated with lower CLAD-free survival of LTRs, independently of the presence of DSA at 3 months (HR 1.8, *p* = 0.024, 95% CI 1.08–3.03). Our data indicate that FCGR SNPs differentially affect the clinical outcome of LTRs and may be of use to stratify patients at higher risk of experiencing graft rejection. Furthermore, these data suggest that in the LTx setting, specific mechanisms of humoral alloreactivity, which cannot be solely explained by the complement and CD16-mediated pathogenic effects of DSA, may be involved in the development of acute and chronic lung allograft rejection.

## Introduction

Lung transplantationi (LTx) remains a challenging therapeutic option for patients with end-stage pulmonary disease. Significant improvement in immunosuppressive strategies has led to decreased lung allograft loss in the early post-transplant period. When compared to other solid organ transplantation settings, LTx remains associated with the lowest survival rates with a median survival of 6 years after transplant (1). Chronic lung allograft dysfunction (CLAD) is the main cause of chronic lung allograft rejection and is characterized by an irreversible loss of lung function associated with a high prevalence of complications such as bronchiolitis obliterans syndrome (BOS) and restrictive allograft syndrome (RAS) (2). Factors that relate to the HLA mismatch, to the graft procedure (ischemia, unilateral or bilateral surgery) and initial lung disease can lead to highly variable levels of recipient immune response to the lung allograft. Inflammatory biomarkers and antibodies, occurrence of acute cellular rejection (ACR), infections/colonization, auto-immunity, and air pollution have been analyzed for their potential predictive value in anticipating development of immunological responses associated with CLAD. Toll receptors, pro-inflammatory cytokines and non-classical HLA molecules, i.e., HLA-G and HLA-E polymorphisms or haplotypes, have been associated with CLAD (3–9), but the underlying mechanisms involved in this devastating outcome of LTx in a given recipient are still poorly understood. Antibody-mediated rejection (ABMR) has been associated with a higher incidence of chronic lung allograft dysfunction (CLAD) and mortality after LTx but the specific mechanisms and histological or immunological biomarkers that allow to define the clinical ABMR entity still await further comprehension (10). Detection of preexisting or development of *de novo* donor-specific human leukocyte antigen (HLA) alloantibodies (DSAs) have been extensively investigated for their potential value as biomarkers of humoral responses that may predict adverse outcome of LTx.

Various studies suggest that the pre-transplant detection of circulating DSA is not associated with an increased risk of developing CLAD or related death if prospective cross-match testing was negative (11, 12). The pretransplant detection of DSA and antibodies directed againd non-HLA antigens such as angiotensin type 1 receptor (AT1R) and endothelin type A receptor (ETAR) is reported to have a negative impact on lung transplant outcome (13).

Early detection of *de novo* DSA detected at 1 month after LTx has been significantly associated with a worse outcome (14). Multiplex solid phase single antigen bead assay (SAFB) detection of *de novo* DSAs, notably anti-HLA DQ DSA, have also been associated with acute cellular rejection (ACR) (15) CLAD (16–18). The presence of circulating DSA at the time of lung allograft biopsy has been identified as a risk factor for graft loss (19).

Although there is more and more direct and indirect evidence regarding the role of *de novo* DSA in CLAD occurrence, the mechanisms that sustain the variable toxicity of these anti-HLA antibodies to the lung allograft are still unclear. One of the well-known cytotoxic mechanisms of DSA occurs through the IgG-mediated activation of the complement cascade that results in C4d deposition within the graft. C4d staining in lung allograft biopsies is considered as a diagnosis criteria for ABMR (10, 20–28). However, although recent evidence suggests that detection of complement-binding DSA can be associated with lung allograft failure (29), the predictive value of complement-binding DSA on LTx clinical outcome remains to be firmly demonstrated.

IgG antibodies can also activate the immune system through ligation of functional Fc gamma receptors (FcyRs). Since these receptors are expressed by a variety of immune cells, including B cells, natural killer cells, platelets, dendritic cells and macrophages, FcR receptors constitute a major checkpoint that regulates the intensity of auto- and allo-immune responsiveness of the host in response to infectious and humoral threats (30, 31). As infections and the immune alloreactivity of the recipient toward the transplant remain a leading cause of graft rejection and death during the first year after LTx, there is a need for biomarkers that may improve the monitoring of early humoral responses in LTRs (32–34) and open therapeutic perspectives to dampen antibody-driven inflammation in immunized patients (35, 36).

Polymorphisms of FcγRIIA (*FCGR2A* [131R/H], rs1801274) FcγRIIIA (*FCGR3A* [158V/F], rs396991) genes, that respectively, encode for the CD32 and CD16 receptors for the Fc segment of IgG, have an impact on the level of expression and immune function of these activating FcγRs. Various reports have highlighted the clinical relevance of these SNPs in controlling the host immune response to monoclonal antibody therapy. The presence of a histidine (H) rather than an arginine (R) at position 131 results in higher affinity for IgG1 and IgG2. The *FCGR2A* [131H/H] genotype has been associated with higher efficacy of Rituximab-mediated B cell depletion strategies before transplant of ABO-incompatible organs. This *FCGR2A* [131H/H] SNP has also been identified as a susceptibility marker associated with the severity of community-acquired pneumonia (37).Genetic variation in the *FCGR2A* gene has also been shown to be associated with an increased prevalence of invasive pneumococcal diseases and respiratory infections after LTx (38, 39), regardless of the site of pneumococcal infection (40). Pro-inflammatory effects associated with the *FCGR2A* [131R/R] genotype have also been reported as a consequence of activating signals that result from the dynamic interactions between CD32 and its cognate C-reactive protein (CRP) and immunoglobulin ligands.

The clinical relevance of CD16 inflammatory pathways in antibody-mediated rejection of kidney and heart allografts has also been illuminated by studies that deciphered the molecular landscape of immune and endothelial cell transcripts that associate with ABMR lesions evaluated in allograft biopsies (41–43). Ligation of the Fc domain of IgG has also been identified as a mechanism that allows CD16-dependant clearance of viral pathogens by immune NK cells (44). Recent studies suggest that the Fc fragment of DSA can indeed exert its adverse cytotoxic and inflammatory effects though a complement-independent mechanism that relies on level of expression of the NK-cell surface CD16 receptor and its affinity for the Fc fragment of alloantibodies. The intensity of host antibody dependent cell cytotoxicity (ADCC) is known to be conditioned by the capacity of NK effector cells to form conjugates with antibody-coated allogeneic cells, which is in part influenced by the *FCGR3A* [158V/F] genotype (45–47). We and others have recently shown that polymorphic variation in the *FCGR3A* genes is also likely to affect the pathogenic effects of IgG alloantibodies by controlling the level of the CD16 dependent recipient's immunological cytotoxic responses to allogeneic donor cells exposed to chronic alloantibody threat (35, 48–51).

Genotypic variation in the *FCGR3A* receptor can thus impact the strength of FcR-dependent ADCC responses of NK cells and regulate their capacity to secrete inflammatory cytokines and release CD107a/Lamp1^+^ cytotoxic granules containing perforin and granzyme (50, 52, 53). The inter-individual variability in the functional CD16 receptor-dependent engagement by the Fc fragment of alloantibodies has been shown to condition the intensity of antibody-dependent cellular cytotoxicity (ADCC) of NK cells and the response to IgG immunotherapy.

The strength of FcR-mediated ADCC can also be affected by the isotype and glycosylation status of alloantibodies. The IgG1 and IgG3 isoforms have higher affinity for the CD16 *FCGR3A* [158V/V] variant when compared to *FCGR3A* [158F/F], thereby spurring increased effector cell activity in response to these IgG subclasses. We have shown that this high-affinity homozygous *FCGR3A* [158V/V] genotype is an independent predictor of cardiac allograft vasculopathy (48) and may be a clinically relevant underlying mechanism that sustains the level of DSA-mediated allograft injury (48–50). The recent evidence that the NK cell infiltration of the graft can predict kidney graft failure also sustains the clinical relevance of these NK cell mediated mechanisms of allograft injury (54).

Considering the central contribution of polymorphisms affecting FcγRIIA and FcγRIIIA genes and receptor function in the individual shaping of antibody-mediated inflammatory and cytotoxic alloimmune responses of the recipient, deciphering the mechanisms and FcγR susceptibility profiles that may be associated with LTx outcome is necessary to optimize risk stratification (8).

This study thus aimed to investigate whether FcγR polymorphisms may be linked to the pathogenic mechanisms of DSA toxicity and be associated with adverse clinical complications that impair patient and allograft outcome after LTx.

## Materials and Methods

### Participants and Study Design

We conducted a retrospective single-center study enrolling 158 adult patients who underwent lung transplants (LTx) at the Marseille Lung Transplant Center between December 2006 and December 2013. All patients from the French cohort (COLT, *Cohort in Lung Transplantation*, l'Institut du Thorax, INSERM UMR1087/CNRS UMR 6291, CNIL 911142) were recruited in this study. All subjects gave written informed consent in accordance with the Declaration of Helsinki. A group of 184 healthy unrelated volunteer French bone marrow donors were also recruited to constitute a control cohort, allowing the analysis of the FcγR genotype. Blood donations were collected in the “Etablissement Francais du Sang,” in accordance with BSL-2 practices. A medical interview was carried out prior to blood donation to exclude donors with medical contraindications. This study was carried out in accordance with the French Public Health Code (art L1221-1), approved by an institutional ethics committee and conducted in compliance with the Good Clinical Practice Guidelines declaration of Helsinki and Istanbul.

### Post-transplant Clinical Management

All recipients received a similar standardized immunosuppressive regimen in accordance with our institutional protocols. Induction therapy consisted of intravenous administration (IV) of rabbit anti-thymocyte globulins (rATG, Pasteur Merieux, Lyon, France) given for the first 3 post-operative days [except when daily lymphocyte count was below 200/mm^3^, and when there were cytomegalovirus (CMV) and/or EBV mismatches (i.e., seronegative recipient and seropositive donor)] and high dose methylprednisolone (6 mg/kg/d Day 1, 2 mg/kg/d Day 2 and Day 3, and 1 mg/kg/d thereafter). The standard triple maintenance immunosuppressive regimen consisted of tacrolimus (adjusted to maintain whole blood levels varying between 12 and 14 ng/ml), mycophenolate mofetil in 5 patients (adjusted to a white blood cell count above 4,000 mm^3^), and steroids (prednisone) tapered to 0.25 mg/kg/d over the first 3 months and stopped around 12 months after surgery.

According to CMV recipient positive (R+) and donor positive/recipient negative (D+/R–) status, patients received CMV prophylaxis with IV ganciclovir switch to oral valganciclovir.

Postoperatively, recipients received prophylactic or preemptive anti-infection treatment (antibiotic, antiviral, and antifungal therapies) according to their preoperative and/or concomitant infectious status.

Recipients had regular visits to the transplant center for clinical radiological and functional evaluation. At our institution, surveillance transbronchial biopsies are routinely performed at the end of the first month, or earlier if clinically indicated. All transbronchial biopsies were graded for ACR (A grade) and lymphocytic bronchiolitis (B grade) (LB) by lung transplant pathologists. ACR and LB were defined, respectively, as perivascular or peribronchial mononuclear inflammation according to the International Society for Heart and Lung Transplantation (ISHLT) criteria. Histologic appraisal of ACR and LB was conducted in accordance with accepted ISHLT standards in terms of the minimum number of biopsy samples and exclusion of opportunistic infection (27, 28). Pulmonary function tests (PFTs) were routinely conducted at our center on a monthly basis for the first 12 post-operative months, at M2 intervals in the second year and at M3 intervals thereafter. The baseline FEV_1_ value was calculated as the average of the 2 best FEV_1_ values at least 2 measure gap. Baseline values of total lung capacity (TLC) and FEV_1_/FVC were defined as the average of the 2 measurements obtained at the same time as the best 2 FEV_1_ measurements. Chronic lung allograft dysfunction (CLAD) was defined according to the standardized international criteria (55). The phenotype BOS or RAS was specified according to ISHLT guidelines (56). The following histological patterns compatible with AMR were also analyzed: (i) neutrophil capillaritis and (ii) acute lung injury with or without diffuse alveolar damage and with or without organizing pneumonia.

### HLA Antibody Screening and Identification Protocol

Recipient serum samples from 2006 to 2013 were collected routinely prior to transplant, at the minimum when the patient was placed on the waiting list and before the transplant procedure (D0: Day 0) and serially after LTx (at month 1 and 3). All sera samples obtained from the 158 recipients were further assessed using Luminex single-antigen flow beads (SAFB) to determine antibody specificity using Single Antigen—One Lambda reagents (*LABScreen*® Single Antigen class I or *LABScreen*® Single Antigen class II, One Lambda, Thermo Fisher Scientific, Canoga Park, California, USA) according to the recommendations of the manufacturers. The mean fluorescence intensity (MFI) used the baseline formula proposed by the Fusion^TM^ v 3.2 software. All beads with a normalized MFI threshold >1,000 were considered positive. Since detected circulating alloantibodies were often directed against distinct HLA class I and/or class II antigens, the cumulative mean fluorescence intensity (cMFI) of DSA was calculated as the sum of the MFI for each of the individual DSAs detected. DSA were considered to be HLA antibodies directed against donor HLA antigen. Putative HLA-Cw and -DQA1 DSA were identified in accordance with the conventional linkage disequilibrium that is reported between HLA-B and HLA-C loci and between HLA-DQB1 and HLA-DQA1 loci, as respectively, described in the French population (57) and by supplementary extensive genotyping of HLA- genes in the recipient. Putative HLA-DR51, -DR52 and -DR53 DSA were determined in accordance with the linkage disequilibrium between DRB3, DRB4, DRB5 loci and DRB1 loci of recipient. The allelic specificities of HLA-Cw, DQA and -DR51, -DR52 and -DR53 DSA were assigned accordingly.

### C1q Detection

All patients with HLA antibodies were tested for the presence of C1q. The binding level was determined by the C1qScreen™ assay per manufacturer instructions (One Lambda, Thermo Fisher Scientific, Canoga Park, California, USA). Fluorescence intensity was measured using Luminex-based LABScan™ 100 flow analyzer. C1q specific antibody specificity and binding levels were analyzed and determined through the Fusion^TM^ v 3.2 software. C1q MFI > 1,000 was considered positive.

### *FCGR2A* and *FCGR3A* Genotyping

The detection of single nucleotide polymorphisms (SNPs) allowed for the genotyping of *FCGR3A* ([158V/F], rs396991) and *FCGR2A* ([131R/H], rs1801274) as described (5, 48) using the SNAPSHOT technique. Genomic DNA was extracted from a 200-μl whole blood sample using the QIAmp Blood DNA Mini kit (Qiagen, Courtaboeuf, France) according to manufacturer instructions. A previously described homemade primer extension method (58) was used to simultaneously analyze the SNPs of the FcγR genes. The forward and reverse primers sequences were, respectively: *FCGR3A*-F 5′ TCCTAATAGGTTTGGCAGTG 3′ and *FCGR3A*-R 5′ AAATGTTCAGAGATGCTGCT 3′, *FCGR2A*-F 5′ CCAGGAGGGAGAAACCATC 3′ and *FCGR2A*-R 5′ CTCTTCTCCCCTCCCTACAT 3′. The extension primers are, respectively: *FCGR3A*_176-F: 30T-CCTACTTCTGCAGGGGGCTT and *FCGR2A*_166F 57T-CCAAAAGCCACACTCAAAGA. Data were analyzed using GeneMapper 4.0 with specific detection parameters. Using an in-house computer program, output files (.txt) exported from GeneMapper 4.0 were automatically formatted into files readable by the “Phenotype” application of the Gene[Rate] computer tool package (http://geneva.unige.ch/generate). For each different allele obtained, PCR products were sequenced on both strands using the BigDye Terminator v1.1 Sequencing Kit (Applied Biosystems) and analyzed on an automated fluorescence-based ABI PRISM 3130 XL genetic analyzer according to manufacturer protocol. FcR polymorphisms at these 4 loci were analyzed for allele frequencies, or homozygous or heterozygous allelic combination of FcγR alleles defining the corresponding genotype.

### HLA Genotyping

Recipients and deceased donors were genotyped for low resolution HLA-A, -B, -DRB1, and -DQB1 loci by LABType® SSO (One Lambda, Thermo Fisher Scientific, Canoga Park, California, USA) according to the manufacturer's specifications and the retrieved output was analyzed for allele identification using HLA Fusion^TM^ v 1.2.1. Software (One Lambda, Thermo Fisher Scientific, Canoga Park, California, USA).

### Phenotypic Analysis of Antibody-Dependent NK Cell Activation

Evaluation of the CD16-dependent alloreactive potential of DSA was assessed using the previously described NK cell humoral activation test (NK-CHAT) (50). The level of serum-induced CD16 engagement and the degranulating potential of NK cells was assessed by flow cytometry analysis of the level of CD16 cell surface expression within the CD3^−^CD56^+^ NK cell compartment, gated within PBMC effector cells after exposure to target B cells that had been precoated in the presence of LTRs or control sera. Effector cells used in the standardized assay were prepared from a healthy donor displaying the *FCGR3A* [158V/V] encoding the high-affinity CD16 variant. The HLA typing of the two B cell lines used as targets were A2/A2, B44/B56, Cw1/Cw5, DR1/DR4, DR53, DQ5/DQ7 and A3/A3 B7/B35, Cw4/Cw5, DR10/DR15, DR51, DQ5/DQ6). Sera were selected for the presence of DSA recognizing a limited set of HLA antigens expressed by EBV target cell lines. Briefly, 500,000 target B cells (B-EBV cell lines expressing the cognate DSA target alloantigens detected in LTR) were incubated with control (CTL) unsensitized human male AB serum (Lonza) to block FcR, then rinsed and incubated for 15 min in the presence of 50% LTR serum [or CTL serum supplemented or not with 10 γg/ml of Rituximab IgG (positive control)] and then rinsed to remove unbound antibodies. Incubation of effector PBMC and pre-coated allogeneic B-EBV cell targets (1:1 ratio) was performed for 3 h at 37°C in presence of GolgiStop (Becton Dickinson 554724) and CD107-PC5 (Becton Dickinson 555802). Cells were then washed and labeled with CD3-ECD (Beckman Coulter A07748), CD16-PE (Beckman Coulter A07766), CD56-PC7 (Beckman Coulter A21692) for 15 min at room temperature protected from light. After 1 wash step, cells were resuspended in 500 μl PBS 2% SVF. Data acquisition and analysis was performed on a Beckman Coulter Navios cytometer. The mean fluorescence intensity of CD16 and percent of NK cells expressing Lamp1/CD107a expression was analyzed within the CD3-CD56+ NK cell subset. The NK-CHAT CD16 down regulation index (CD16DRI) was evaluated as a ratio between CD16 MFI measured on NK cells incubated with target cells in presence of effector cell autologous CTL serum/CD16 MFI of NK cells incubated with target cells and recipient serum to be tested. CD16DRI = (MFI CD16 control serum)/(MFI CD16 test serum). Alternatively, when serum at time of LTx was available for the assay, the baseline CD16 expression was evaluated in reference to the DSA negative serum collected at time of LTx. Sera containing HLA-DQ7 DSA obtained from kidney transplant recipients (KTR) with antibody-mediated rejection (ABMR) at time of diagnosis, previously described to induce high levels of CD16-dependent NK cell alloreactivity, were introduced as positive controls of the experiments, indexing the CD16 down regulation induced by LTx sera.

### Statistical Analyses

Variables used to perform univariate and multivariable analyses included preoperative donor variables (donor age, sex, CMV status, HLA typing), preoperative donor–recipient matching parameters (age, sex, CMV status, and HLA mismatch), operative variables such as ischemia time, type of procedure (single vs. bilateral LTx) and preoperative and post-operative recipient variables (initial lung disease, HLA typing, pre- and post-transplant immunization status, occurrence of AR BOS, RAS, CLAD, or infectious bacterial episodes occurring during the first year post-transplant), and notably during the early post-Tx period at months 1 (M1) and 3 (M3) after LTx (Table 1).

**Table 1:** Demographic and Clinical characteristics of LTRs.

**Recipient, *n***	**158**
Recipient age at LTx (years), median(25–75)	42 (30–54)
Recipient Age ≥ 50, *n* (%)	54 (34)
Donor Age, median (25–75)	43 (29–54)
Male Gender, *n* (%)	83 (53)
**Native Lung Disease**	
Cystic Fibrosis, *n* (%)	59 (37)
Fibrosis, *n* (%)	35 (22)
Emphysema, *n* (%)	46 (29)
Other, *n* (%)	18 (12)
**Transplantation type**	
Bilateral LTx, *n* (%)	118 (74)
Single LTx, *n* (%)	36 (24)
Other, *n* (%)	4 (2)
**CMV Risk**	
CMV R- (%)	50
CMV Mismatch D+R- (%)	21
**Immunization Status**	
HLA Mismatch, median (25–75)	7 (6–7)
DSA M1, *n*	49
MFI DSAM1, median (25–75)	13,000 (5,500–18,750)
DSA M3, *n*	27
MFI DSA M3, median (25–75)	7,100 (3,500–13,500)
M1 DSA persisting at M3, *n*	21
C1q DSAM1, *n*	20
C1q DSAM3, *n*	7
**Bacterial Infections**	
M1, *n*	20
M3, *n*	11
First year, *n*	35
**Transplant Outcome**	
Time follow up post-LTx (months), median (25–75)	38.5 (23–62)
**Rejection events**	
Biopsy proven Acute rejection Day 0-M3, *n* (%)	41 (28)
Biopsy proven Acute rejection first year, *n* (%)	53 (35)
Time before Acute Rejection event (Days), median (25–75)	42 (22–179)
CLAD, *n*	40 (25)
Time before CLAD (months), median (25–75)	22 (13–32)
BOS, *n* (%)	35 (22)
RAS, *n* (%)	5 (3)
Allograft and Patients' Survival	
CLAD- or Graft failure-free Survival, *n* (%)	71 (45)
Death, *n* (%)	64 (40)
Time before Death (months), median (25–75)	13 (1.8–35)
Death or Graft failure with 2^*nd*^ LTx, *n* (%)	68 (43)

*Data are presented as a number and %, or median (25–75 Interquartile ranges). BOS, Bronchiolitis obliterans syndrome; RAS, restrictive allograft syndrome; CLAD, chronic lung allograft dysfunction; M1 and M3, month 1 and 3 post-LTx*.

Continuous variables were presented as median values and 25–75 interquartile ranges or mean ± sd according to their distribution evaluated using the Agostino & Pearson omnibus normality test performed using GraphPad Prism version 5.00 for Windows (GraphPad Software, La Jolla, California, USA). Categorical variables were presented as percentages. Fisher's exact test and the chi-square test were used to compare categorical data and *t*-tests or Mann Whitney tests were used for the comparison of continuous variables.

The primary endpoints of this study were overall survival (OS) and disease/CLAD-free survival (DFS). OS was defined as the interval between the date of transplant and the last follow-up visit or death. DFS was defined as the time interval from transplant to the first event: either the graft failure or diagnosis of acute rejection or CLAD in living recipients, or the death of the patient. The Kaplan-Meier method was used to estimate overall survival and rejection-free survival. The log-rank test was used to assess the univariate effects on OS and DFS. For all analyses, a 2-sided *p* < 0.05 was considered statistically significant. Variables with a *p* < 0.2 were also considered to construct multivariate regression models. Multivariate analyses were performed using Fine and Gray's proportional hazards regression model. All analyses were performed using IBM SPSS 15.0 software (SPSS Inc., Chicago, IL) and the cmprsk package (developed by Gray, June 2001) on R2.3.0 software (http://www.R-project.org).

## Results

### Characteristics of the LTR Cohort

The demographic characteristics and clinical features of the 158 adult LTRs (median age: 42 years, 75 females and 83 males) are summarized in Table 1. Patients received a first LTx for cystic fibrosis (37%), emphysema (29%), pulmonary fibrosis (22%), or another diagnosis (12%). The median follow-up time was 38 months after LTx. Median survival time of the 94 patients who were alive at time of follow-up was 4.2 years. Sixty-four patients (40.5%) died during the study follow-up (median time before death 12.6 months, 25–75 interquartile range: 1.8–35.5 years) and 5 patients experienced CLAD-associated graft failure with indication of a second lung transplant or death. Among deceased patients, 32 patients died during the first year post-LTx, among which 12 patients died during the first month post-LTx and 9 between the first and third months post-LTx. Acute rejection diagnosis was confirmed in the lung transplant biopsy of 53 patients (36%), the first rejection episode occurring before the third month in 41 LTRs (occurring before the fourth month in 24 LTRs and during the third month and the twelfth month post-LTx in 11 LTRs). The occurrence of acute rejection could be associated with the presence of DSA in 49% of these patients (*n* = 20). During the study period, 40 LTRs (25%) developed CLAD, of which 35 (87%) had BOS and 5 (12%) had RAS. Seventeen of the patients with a CLAD diagnosis did not survive and 5 were considered for a second transplant procedure. CLAD-free survival of the graft was observed in 71 patients (45%) with a median disease-free survival time of 4.2 years (25–75 percentile: 3.1–5.8).

### Distribution of *FCGR3A* and *FCGR2A* Polymorphisms

**Figure 1:**
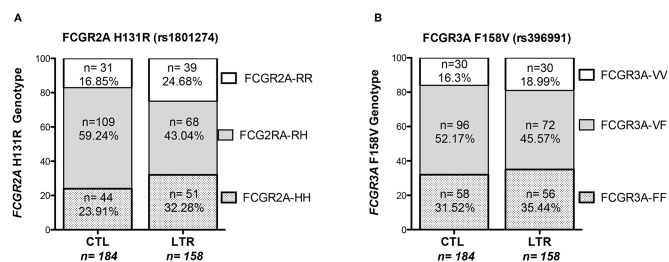
Distribution of *FCGR3A* and *FCGR2A* genotypes in LTRs and CTLs. The proportion of the *FCGR2A* (left Panel, **A**) and *FCGR3A* (right panel, **B**) genotypes resulting from the SNP allelic combination were analyzed in a cohort of 158 LTRs and 184 CTLs recruited in southern France.

Genotyping of the *FCGR2A* and *FCGR3A* polymorphisms was performed in 158 LTRs and analyzed in reference to a control cohort of 184 healthy donors (CTLs) (Figure 1). The *FCGR2A*-H allele was detected in 75.3% of LTRs and 83.1% of CTLs (*p* = 0.0734) while the -R allele was detected in 67.7% of LTRs and in 76% of CTLs (*p* = 0.0851). Although the distribution of *FCGR2A* [131R/R] and *FCGR2A* [131H/H] was not significantly altered in LTRs, the frequency of *FCGR2A* [131H/R] was found to be significantly lower in LTRs analyzed in reference to CTLs (chi-square *p* = 0.0028). The distribution of *FCGR3A* alleles did not significantly differ between patients and controls; presence of the -F allele was detected in 81% of LTRs and in 83.7% of CTLs while the -V allele distribution was in 64.6% of LTRs and in 68.5% in CTLs. The genotype frequencies of the various genotype combinations resulting from analysis of these SNPs in the LTR and CTL cohorts are illustrated in Figure 1. The frequency of the *FCGR3A* [158F/F] genotype was positively correlated with the *FCGR2A* [131R/R] genotype in CTLs and in LTRs (*p* = 0.008 and *p* < 0.0001, respectively). Presence of the *FCGR2A* [131R/R] was thus significantly associated with lower rates of patients with the *FCGR3A* [158V/V] good responder genotypes in LTRs (*p* = 0.038), but this inverse correlation did not reach significance in the control cohort (Table 2). Analysis of the groups stratified according for FCGR SNPs did not reveal any significant difference regarding the age of the LTR and type of underlying initial lung disease. The *FCGR2A* [131H/R] SNP with was found to be associated with recipient gender (*p* = 0.029). The *FCGR2A* [131H/H] genotype was observed to be significantly more common in women LTRs (32 out of 51, *p* = 0.008).

**Table 2:** Association between the *FCGR2A* [131R/R] and FCGR3A [158V/F] genotypes observed in LTRs and CTLs.

		***FCGR2A*** **genotype LTR**			
		**HH** ***n* = 51**	**HR** ***n* = 68**	**RR** ***n* = 39**	***p-*value FCGR2A-RR vs.** **FCGR3A**
FCGR3A genotype	FF, *n* = 56	8	24	24	*p <* 0.001
	VF, *n* = 72	30	30	12	*p =* 0.032
	VV, *n* = 30	13	14	3	*p =* 0.038
		***FCGR2A*** **genotype CTL**			
		**HH** ***n*** **=** **44**	**HR** ***n*** **=** **109**	**RR** ***n*****=** **31**	***p-*****value FCGR2A-RR vs.** **FCGR3A**
FCGR3A genotype	FF, *n* = 58	8	34	16	*P =* 0.008
	VF, *n* = 96	24	59	13	*p =* 0.211
	VV, *n* = 30	12	16	2	*p =* 0.103

### DSA Immunization During the First 3 Months Following Lung Transplant Is Associated With FCGR Polymorphism

**Figure 2:**
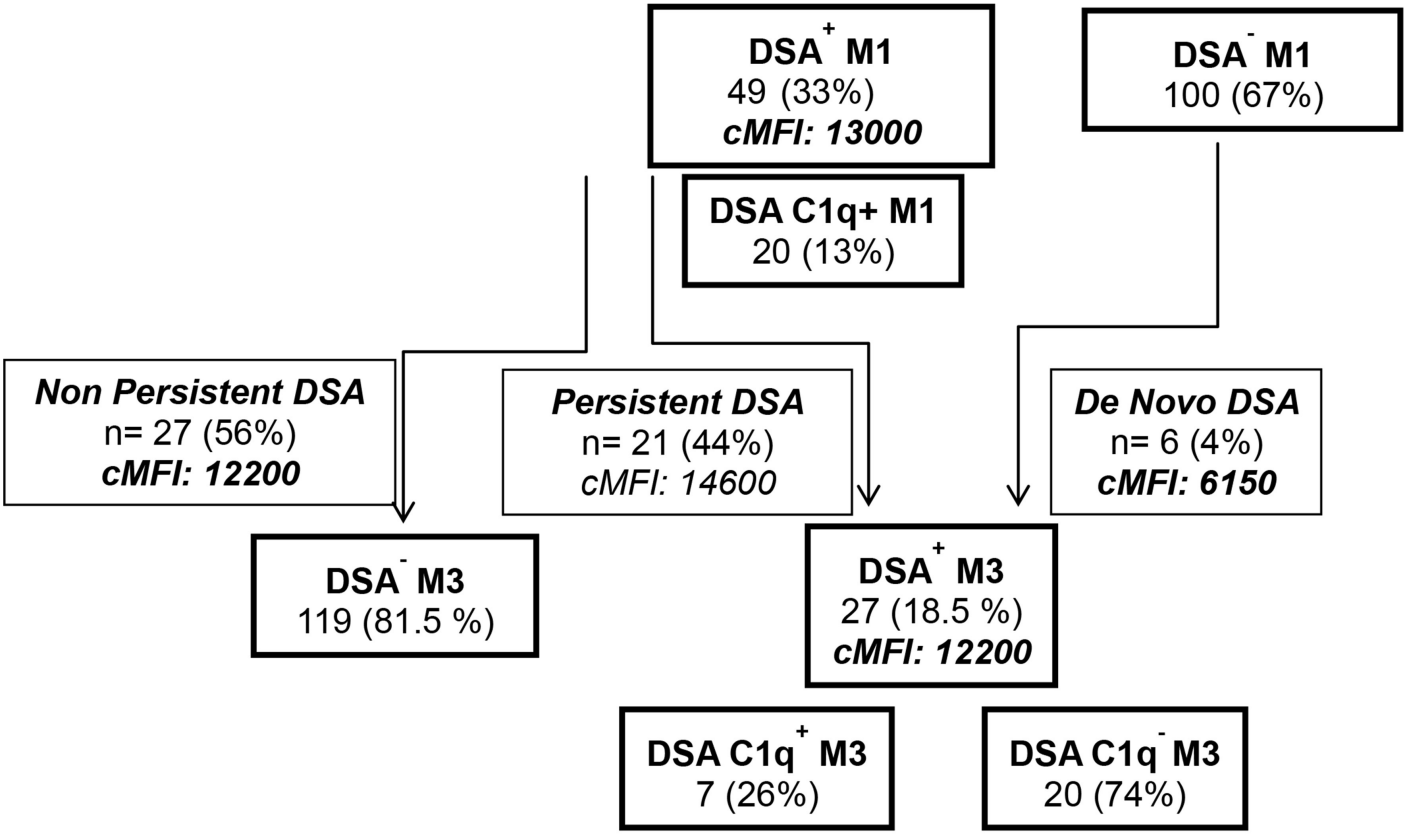
Study flow chart analysis of the detection by single antigen flow bead Luminex Assay of *de novo* DSA and C1q binding activity of DSA at M1 and at M3 post-LTx.

During the first 3 months post-transplant, 55 patients developed *de novo* circulating DSA. During the first month post-LTx (M1), DSA developed in 49 LTRs (12 HLA class I, 20 HLA class II, and 17 HLA class I and II). The median MFI of DSA detected at M1 was 7,000 for HLA Class I DSA and 12,100 for HLA Class II DSA and 12 patients (8%) exhibited high cMFI values of DSA (over the 18,750 threshold corresponding to the 75 percentile value observed for MFI analyzed at M1) (Table 1). Circulating DSA could be detected in 27 LTRs analyzed at month 3 (M3) post-LTx (11 HLA class I, 13 HLA class II and 3 HLA class I and II) (Figure 2). The development of *de novo* DSA was observed at M3 in 6 LTRs with no detectable DSA at M1 (4 HLA class I, 1 HLA class II and 1 HLA Class I and Class II). The median MFI values of DSA detected at M1 and at M3 were, respectively, 13,000 and 7,100 (Table 1, Figure 2). DSA detected at M1 persisted at M3 in 21 LTRs (44% of DSA-positive MTR at M1) and 4 of these M3 DSA were also detectable 1 year post-transplant. HLA-DQ DSA were the only HLA class II persisting at M3. The median cMFI level of DSA detected at M1 and persisting at M3 (14,600, 25–75 percentile, 7,000–23,250) could not be linked to their persistence at M3 and did not significantly differ from the cMFI of DSA observed at M1 but undetectable at M3 (median MFI: 12,200, 25–75 percentile 4,000–16,000). Circulating DSA directed against DQ specificities were detected in 33 LTRs at M1 (median DQ-DSA MFI: 12,000 25–75 percentile 7,600–14,500) and in 14 LTRs at M3 (median 12,000, 5,000–15,850, 11 HLA-DQ DSA detected at M1 persist at M3 while 3 of the 14 HLA-DQ DSA detected at M3 were *de novo* DSA). C1q Binding DSA at M1 were detected in 20 LTRs (2 HLA class I, 15 HLA class II, and 3 HLA class I and II) with a median cMFI of C1q-binding DSA detected at M1 of 23,000 (25–75 percentile 6,300–30,000). Seven of the 27 DSA detected at M3 were found to bind C1q (1 HLA class I, 5 class II HLA DSA and 1 HLA class I and class II), with a median cMFI of 17,150 (25–75 percentile 7,000–31,000). All C1q binding DSA detected at M3 were also detected at M1.

The *FCGR2A* [131R/R] genotype was also to found to correlate with the detection of circulating DSA at M3 (*p* = 0.011) and notably to the development of *de novo* DSA between the first and second months post-transplant (4 out of the 6 *de novo* DSA detected at M3 were genotyped as *FCGR2A* [131R/R], *p* = 0.017). *FCGR3A* [158V/V] tends to be inversely associated with the persistence of DSA at M3 (*p* = 0.07, Table 3), as only 1 out of the 21 patients with persistent DSA at M3 was genotyped as *FCGR3A* [158V/V]. The *FCGR3A*-F allele was significantly associated with the detection of circulating DSA at M3 (26 out of 27 LTRs with detectable circulating DSA at M3 were positive for the *FCGR3A*-F allele, *p* = 0.024) (Table 3).

**Table 3:** Patients immunization characteristics according to the *FCGR2A* and *FCGR3A* genotypes.

	***FCGR2A***			**FCGR3A**		
	**HH/RH**	**RR**	***p*-value**	**FF/VF**	**VV**	***p*-value**
*n = 158*	119	39		128	30	
DSA M1, *n* = 49	33	16	0.161^t^	40	9	0.926, ns
Median MFI DSA M1	13,000	13,500	0.639, ns	12,500	13,800	0.477, ns
C1q DSA M1, *n* = 24	18	6	0.919, ns	19	5	0.869, ns
DSA M3, *n* = 27	15	12	0.011	26	1	0.024
DQ DSA M3, *n* = 14	7	7	0.022	13	1	0.225, ns
M1 DSA persisting at M3, *n* = 21	13	8	0.146^t^	20	1	0.07^t^
Median MFI DSA M3	6,300	9,500	0.494, ns	7,500	4,500	NA
C1q DSA M3, *n* = 7	5	2	0.840, ns	7	0	0.187^t^

Continuous variables are expressed as median (25–75 percentile ranges). p-values of the statistical tests comparing groups (Chi-2 or non-parametric Mann-Whitney test) were considered as significant when p-values were < 0.05, tendency (

t *) when p > 0.05 and < 0.2 and non-significant (ns) when p > 0.2 or NA when non-applicable. M1 and M3, month 1 and 3 post-LTx*.

### *FcGR3A* and *FcGR2A* Polymorphisms Are Differentially Associated With Acute and Chronic Lung Allograft Rejection

**Figure 3:**
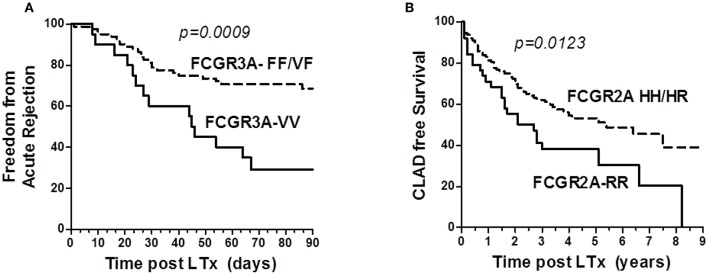
Analysis of the influence of Fc-gamma Receptor polymorphisms on Rejection Free survival. **(A)** Impact of *FCGR3A* genotype on freedom from acute lung rejection. Kaplan–Meir Survival analysis links the *FCGR3A* [158V/V] (solid line, *n* = 20) to lower acute-rejection-free survival after 3 months post-LTx in LTRs, when compared with the *FCGR3A* [158V/F] and *FCGR3A* [158F/F] group (dashed line, *n* = 81). LTRs with the *FCGR3A* [158V/V] show an increased rate of acute rejection events occurring during the first 3 months post-LTx. **(B)** Impact of *FCGR2A* genotype on CLAD free survival. Kaplan–Meir Survival analysis links the *FCGR2A* [131R/R] (solid line, *n* = 39) to a lower CLAD-free survival rate in LTRs over time or study follow-up (years, x axis), when compared with the *FCGR2A* [131H/H] and *FCGR2A* [131R/H] group (dashed line, *n* = 119).

The *FCGR3A* [158V/V] was also identified as a susceptibility genotype associated with a higher risk of acute rejection (log rank test for equality of survivor functions *p* = 0.0009, Figure 3A). Multivariate Cox regression analysis also revealed that the susceptibility conferred by the *FCGR3A* [158V/V] genotype (OR 4.8, *p* < 0.0001, 95% CI 2.375–9.607) was independent from the development of DSA at M3 and their C1q binding activity. *FCGR2A* [131R/H] was not observed to affect the rate of acute rejection. The *FCGR2A* [131R/R] genotype was instead associated with the detection of DSA occurring in the absence of acute rejection (*p* = 0.029).

The DSA immunization status of the patient evaluated during the first year post- LTx could not be associated with CLAD. Although the number of observations was small, 4 of the 5 LTRs who developed RAS-associated CLAD were homozygous for the *FCGR3A*–FF allele (*p* = 0.034). While the *FCGR3A* [158V/F] could not be significantly associated with the risk of CLAD, *FCGR2A* [131R/R] was identified as a susceptibility marker associated with lower CLAD-free survival of LTRs (*n* = 87, logrank test for equality of survivor functions *p* = 0.0123, Figure 3B). Multivariate Cox regression models adjusting co-variables identified using univariate analysis further identified *FCGR2A* [131R/R] as an independent susceptibility marker associated with CLAD (OR 2.2; *p* = 0.022, 95CI 1.126–4.435) (Table 4). Multivariate Cox analysis further showed that the *FCGR2A* [131R/R] genotype is significantly associated with the enhanced risk of developing a composite LTx adverse outcomes (CLAD, graft failure or death), independently of other factors such as detection of DSA at M3 or native emphysema lung disease (Table 4).

**Table 4:** Univariate analysis and multivariate Cox regression analysis of risk factors associated with acute or chronic rejection events.

**Risk covariables**	**Univariate analysis *p-value***	**Hazard ratio**	**Std. Err**.	**z**	***P* > z**	**(95% Conf.interval)**		
**Acute Rejection in the first 3 months post-LTx**, ***n=*** **41**								
*FCGR3A* [158 V/V]	0.003	4.8	1.70	4.39	**<0.0001**	**2.375**	**–**	**9.607**
Native Lung Disease Emphysema	0.037	0.4	0.18	−2.05	**0.040**	**0.173**	**–**	**0.961**
M3 C1q Binding DSA	0.009	4.4	2.87	2.28	**0.023**	**1.231**	**–**	**15.789**
Single lung Tx	0.091	2.6	0.96	2.56	**0.010**	**1.251**	**–**	**5.352**
*FCGR3A* [158 V/F]	0.003	4.4	1.54	4.29	**<0.0001**	**2.247**	**–**	**8.761**
Native Lung Disease Emphysema	0.037	0.4	0.17	−2.17	**0.03**	**0.161**	**–**	**0.909**
M3 DSA^+^	Ns (*p* = 0.251)	3.1	1.27	2.82	**0.005**	**1.417**	**–**	**6.917**
Single lung Tx	0.091	2.9	1.12	2.89	**0.004**	**1.419**	**–**	**6.243**
**CLAD**, ***n=40***								
*FCGR2A* [131R/R]	0.185	2.23	0.781	2.3	**0.022**	1.126	–	4.435
Native Lung Disease Emphysema	0.177	2.65	0.900	2.86	**0.004**	1.360	–	5.157
Recipient Gender: Female	0.142	1.62	0.531	1.47	0.141	0.852	–	3.080
Acute Rejection 1st Year	0.050	1.93	0.621	2.03	**0.042**	**1.024**	**–**	**3.625**
**Composite adverse outcome: CLAD, graft loss or death** ***n*** **=** **87**								
*FCGR2A* [131R/R]	0.185^t^	1.8	0.475	2.3	**0.024**	**1.080**	**–**	**3.028**
Native Lung Disease Emphysema	0.177	2.1	0.514	2.95	**0.003**	**1.279**	**–**	**3.439**
M3 DSA	0.075	1.9	0.557	2.44	**0.015**	**1.145**	**–**	**3.080**

*Covariables (risk covariables) used to explain the Lung transplant outcome primary variable (Acute Rejection, CLAD or adverse composite outcome that includes CLAD or Graft loss or death) are listed on the left column. The bold text refers to covariables that retained independent significant p-values in multivariate cox regression models*.

### LTx DSAs Have No Major Impact on CD16-Dependent NK Cell Cytotoxic Activation

Considering our previous finding that identifies the potential value of *FCGR3A* [158V/F] in predicting cardiac allograft vasculopathy (48), we further investigated whether the presence of association of acute rejection events with high affinity *FCGR3A* [158V/V] genotype could be associated with the DSA-mediated pathogenic effects that promote acute rejection mechanisms of lung allograft. Using the NK-cellular humoral activation test (NK-CHAT), previously designed to index the level of DSA-mediated engagement of CD16 and unravel potential ADCC-driven pathogenic effects of circulating DSA (41), we therefore aimed to investigate whether circulating DSA detected at M1 or M3 in LTRs have the potential to stimulate NK cell alloreactivity. Evaluation of the DSA-induced down regulation of NK cell CD16 expression (CD16 Down Regulation Index or CD16DRI) was performed in a standardized NK-CHAT assay analyzing alloreactivity of NK cells toward serum-coated CD20^+^ B lymphocytes expressing the cognate HLA target alloantigen, using 23 serum samples obtained from 20 distinct LTRs (Table 5). Thirteen sera were collected at M1 and ten at M3. Four sera had detectable DSA that persisted at M3. Seven DSA at M1 and Four DSA at M3 were C1q positive.

**Table 5:** NK-CHAT: Evaluation of DSA reactivity toward B cell targets expressed in sera collected from LTR and KTR, used as positive controls for NK-CHAT.

**LTR**	**Serum Time post-LTx**	**DSA HLA class I/II**	**MFI DSA**	**MFI C1q DSA**	**Serum induced CD107 up regulation (CD107URI)**	**Serum induced CD16 down regulation index (CD16DRI)**	**CLAD**	**AR**	**Death**
LTR 1	M1	Class I	A3:8,500 B7:8,500	A3:14,000 B7: > 15,000	1	1	na	1	1
LTR 2	M1	Class I	A2:7,000 B44:3,000	A2: 3,000 B44: 10,000	1	1	1	1	0
LTR 3	M1	Class I	A2: 3,200	Negative	1	1	1	1	0
LTR 4	M1	Class I	A3: 2,300	Negative	1	1	0	0	0
LTR 5	M3	Class I	B7: 3,000	Negative	1.6	1	0	1	0
LTR 6	M1	Class I and II	A2: 3,500 DQ7: 13,000	DQ7: 10,000	0.4	1	0	0	0
	M3	Class II	DQ7: 9,000	Negative	0.5	1	0	0	0
LTR 7	M1	Class II	DQ2:10,000 DR53:3,000	Negative	3.3	1.2	na	0	1
LTR 8	M1	Class II	DQ5: 9,500	Negative	1.6	1	1	1	1
LTR 9	M1	Class II	DQ6: 10,000	Negative	1.4	1.2	0	0	0
LTR 10	M1	Class II	DQ7 > 15,000	DQ7 > 15,000	0.9	1.3	0	0	0
LTR 11	M1	Class II	DQ7:11,000 DQ9:4,000	DQ7:11,500 DQ9:11,500	0.9	1	1	0	1
LTR 12	M1	Class II	DQ7: 13,000	Negative	1.4	1.1	0	0	0
LTR 13	M3	Class II	DQ5: 14,000	DQ5: 9,000	0.9	1.4	0	0	0
LTR 14	M3	Class II	DQ5: 13,000	Negative	<1	<1	na	0	**1**
LTR 15	M3	Class II	DQ5 4,500	Negative	<1	<1	0	0	0
LTR 16	M3	Class II	DR53: 3,000	Negative	1	1	0	0	0
LTR 17	M3		DQ7: 13,400	DQ7 11,000	1	1			
LTR 18	M3	Class II	DQ7: 16,000	DQ7, 14,000	3	2.1	na	0	1
LTR 19	M1	Class II	DQ5:7,500 DQ6:6,000	DQ5: 7,000 DQ6: 6,500	1.2	<1	0	1	0
	M3	Class II	DQ5: 3,500	Negative	<1	<1			
LTR 20	M1	Class II	DQ7: 9,000	DQ7: 6,000	1.5	1.9	0	0	0
	M3	Class II	DQ7: 12,500	DQ7: 9,000	1.4	1.9			
KTR 1 ABMR	At time of ABMR diagnoss	Class II	DQ7: 9,000	nt	6.5	29	na	na	na
KTR 2 ABMR		Class II	DQ7: 13,000	nt	7	42	na	na	na

*AR, acute rejection; na, not applicable; nt, non-tested*.

**Figure 4:**
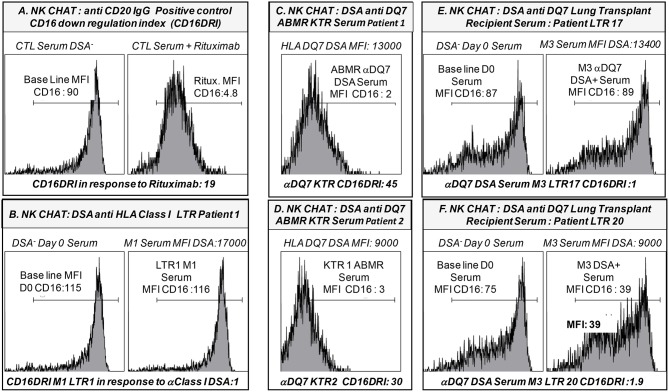
Representative illustration of the NK-Cellular Humoral test (NK-CHAT) used to evaluate serum- and DSA- induced NK cell activation toward B cell lines expressing cognate HLA class I or HLA-DQ7 donor-specific allo-antigens. **(A)** PBMC effectors obtained from *FCGR3A* [158 V/V] CTL were exposed to B-EBV cell lines previously coated with DSA-negative serum. This allowed for evaluation of the baseline expression level of CD16 expression (CD16MFI) within the CD3-CD56+CD16+ NK cell compartment when PBMC were exposed to B cell targets. As a positive control indexing the level of IgG-induced CD16 down regulation (CD16DRI), the same B-EBV cell lines were coated in the presence of 10γ/ml Rituximab (anti CD20 IgG) prior to exposure to the effector PBMC. This allowed for calculation of the CD16 down regulation index (CD16DRI: baseline CD16MFI NK cells exposed to B cells coated with CTL DSA-negative serum/CD16 MFI of NK cells exposed to the same B targets pre-coated in presence of Rituximab). In this context, CD16DRI in response to Rituximab = 19, i.e., 90/4.8. **(B)** The CD16 DRI of NK cells was evaluated in response to the serum collected at M1 in one LTR (LTR 1, Table 5) with detectable levels of DSA recognizing the HLA-A3 and -B7 expressed on the B cell targets. Non-immunized serum (DSA-negative) collected from LTR 1 at time of LTx (D0) was used as the reference baseline CD16 MFI value to calculate the CD16DRI. Despite a cumulative MFI of DSA >17,000, DSA detected at M1 in LTR1 failed to induce CD16-dependent NK cell alloreactivity (CD16DRI = 1). **(C)** The CD16 DRI of NK cells was evaluated in response to the serum collected at time of ABMR diagnosis in one kidney transplant recipients (KTR 1, Table 5) with detectable levels of DSA recognizing the HLA-DQ7 (MFI: 13,000) expressed on the B cell targets. The CD16DRI of KTR1 (CD16DRI: 45) was greater than that observed in response to the Rituximab (CD16DRI:19, **A**). **(D)** The CD16 DRI of NK cells was evaluated in response to the serum collected at time of ABMR diagnosis in a second KTR (KTR 2, Table 5) with detectable levels of DSA recognizing the HLA-DQ7 (MFI: 9,000) expressed on the B cell targets. The CD16DRI of KTR1 (CD16DRI: 30) was greater than that observed in response to the Rituximab (CD16DRI:19, **A**). **(E)** The CD16 DRI of NK cells was evaluated in response to the serum collected at M3 (time of acute rejection) in a second LTR (LTR 17, Table 5) with detectable levels of DSA recognizing the HLA- DQ7 (MFI: 13,400) expressed on the B cell targets. Non-immunized serum (DSA-negative) collected from LTR 17 at D0 was used as the reference baseline CD16 MFI value to calculate the CD16DRI (CD16DRI = 1). The MFI DSA of LTR 17 (MFI: 13,400) was similar to the MFI DSA of ABMR KTR 1 serum as illustrated in **(C)**. **(F)** The CD16 DRI of NK cells was evaluated in response to the serum collected at M3 (time of acute rejection) in a third LTR (LTR 20, Table 5) with detectable levels of DSA recognizing the HLA- DQ7 (MFI DSA: 9,000) expressed on the B cell targets. The DSA-negative serum of LTR 20 collected at D0 was used as a baseline CD16 MFI value to calculate the CD16DRI (CD16DRI = 1.9). The MFI DSA of LTR 20 (MFI: 9,000) was similar to the MFI DSA of ABMR KTR 2 serum evaluated in **(D)**.

Surprisingly, the CD16DRI values induced by the DSA^+^ LTx sera were very low when compared to those obtained in response to Rituximab. These CD16 DRI values could not be associated with the intensity of DSA MFI and C1q binding activity of DSA. Furthermore, the CD16DRI of LTRs (LTR 17, 18, and 20) with HLA DQ7 DSA were lower than those obtained of ABMR kidney transplant recipients (KTR) with HLA DQ7 DSA and with similar MFI (Figure 4).

In contrast to the results previously observed in the kidney and heart transplant setting, the association of the *FCGR3A* [158V/V] genotype with acute rejection appears to be independent of the presence of circulating DSA and the DSA MFI levels. In contrast to the KTR serum, NK-CHAT evaluation of the serum of immunized LTRs did not reveal significant alloreactive DSA toxicity and did not allow for determination of their pathogenic potential in eliciting CD16 and NK-cell cytotoxic activation.

### *FCGR2A* Polymorphism Impacts Patients and Allograft Survival

Occurrence of acute and chronic events or infectious episodes was not shown to have a major impact on overall survival in the analyzed cohort.

**Figure 5:**
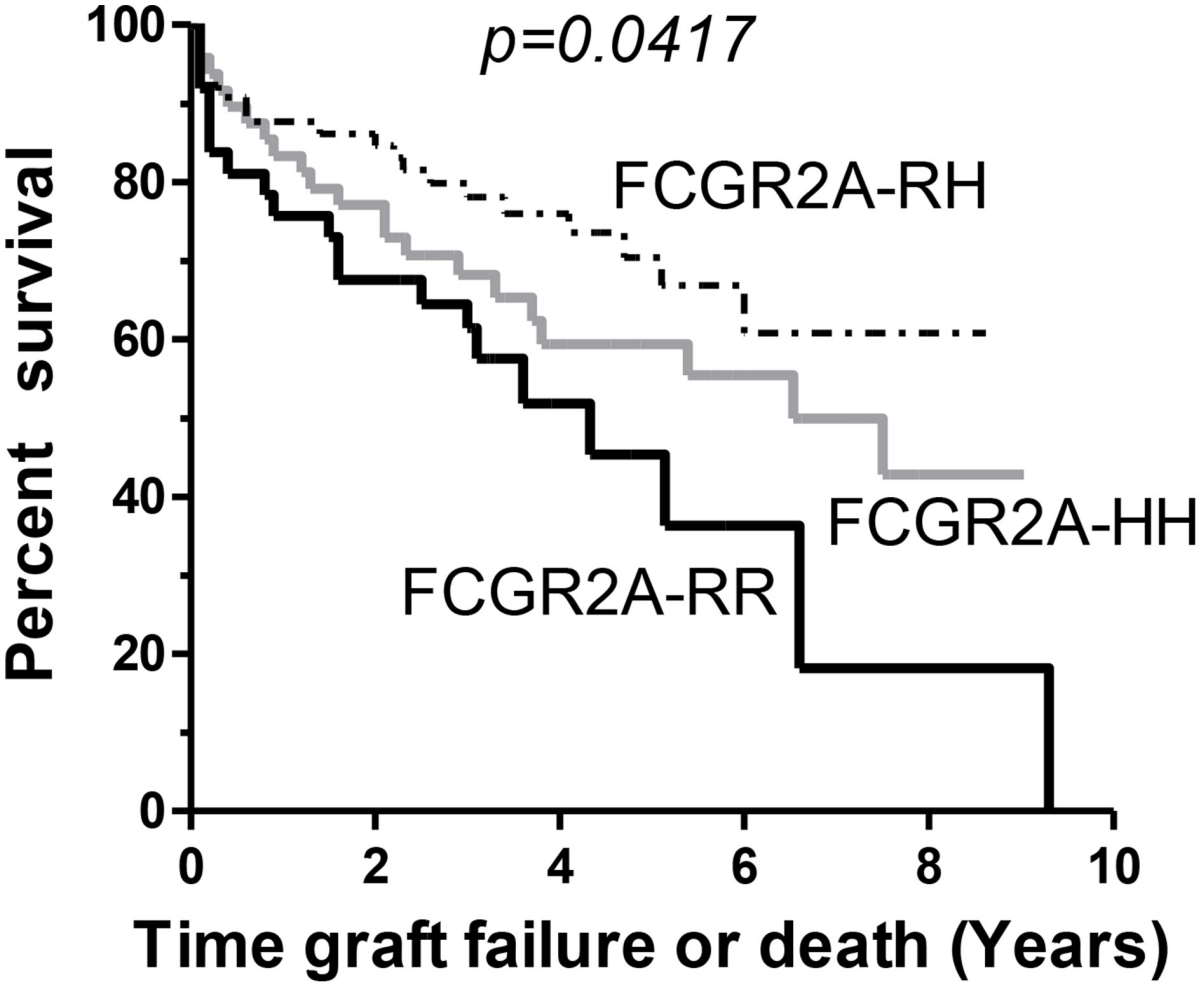
Kaplan-Meir survival analysis of lung allograft survival stratified according to *FCGR2A* [131R/H] genotypes. The *FCGR2A* [131R/R] homozygous genotype (solid black line, *n* = 39) is associated with lower survival rates in LTRs when compared to *FCGR2A* [131H/R] (gray line, *n* = 68) or the *FCGR2A* [131H/H] (dashed line, *n* = 51).

Early development of DSA that persist at M3 (*n* = 21) were associated with lower graft or patient survival rates (*p* = 0.002) and were associated with the presence of the low-affinity F allelic variant of CD16. Persistence of DSA at M3 was thus lower in *FCGR3A* [158V/V] LTRs (*p* = 0.029). Other parameters such as high MFI DSA detected at M1 (>18,750 MFI, 75 percentile value of DSA MFI at M1 post-LTx, *p* = 0.109) or FCRGR2A [131R/R] (*p* = 0.052) tended to be associated with the risk of death. While the FCGR3A genotype could not be associated to overall survival, the FCRGR2A [131R/R] genotype was identified as a risk factor associated with the composite adverse outcome combining graft failure or patient death (*n* = 68, log rank test for equality of survivor functions *p* = 0.0417, Figure 5). Multivariate analysis of variables associated with death from all causes after LTx (*n* = 64, 40.5% of the LTR cohort) retained initial diseases other than cystic fibrosis (*p* = 0.048) DSA with MFI values > to the 75 percentile 18,750 MFI value for DSA detected at M1 as significant risk factors (Table 6). The FCRGR2A [131R/R] genotype was shown to be an independent factor associated with lower overall survival of LTRs using multivariate logistic Cox regression models (HR: 1.8, *p* = 0.047 95% CI: 1.008–3.121, Table 6).

**Table 6:** Univariate analysis and multivariate Cox regression analysis of variables associated with graft loss and patient death post-LTx.

**Covariables**	**Univariate analysis *p-value***	**Hazard ratio**	**Std. Err**.	**z**	**P>z**	**(95% Conf.interval)**		
**Death, All causes**, ***n*** **=** **64**								
*FCGR2A* [131R/R]	0.114	**1.8**	**0.511**	**1.99**	**0.047**	**1.008**	**–**	**3.121**
Native Lung Disease: Cystic Fibrosis	**0.048**	**0.5**	**0.53**	**−2.25**	**0.024**	**0.278**	**–**	**0.915**
MFI DSA M1 > 18,750 (75 percentile)	0.109	**2.7**	**1.123**	**2.36**	**0.018**	**1.184**	**–**	**6.093**
**Graft loss or LTR death**, ***n*** **=** **68**								
*FCGR2A* [131R/R]	**0.052**	**1.85**	**0.552**	**1.99**	**0.047**	**1.008**	**–**	**3.392**
MFI DSA M1 > 18,750 (75 percentile)	0.166	**2.8**	**1.259**	**2.29**	**0.022**	**1.159**	**–**	**6.759**
RAS	**0.009**	**2.8**	**1.385**	**2.08**	**0.037**	**1.062**	**–**	**7.384**
Native Lung Disease: Cystic Fibrosis	**0.034**	0.53	0.181	−1.86	0.063	0.272	–	1.034
DSA at M1 persisting at M3	0.153	1.37	0.550	0.78	0.436	0.622	–	3.009
Bilateral Lung Transplant	0.161	0.92	0.319	−0.24	0.814	0.467	–	1.818

*Covariables (risk covariables) used to explain the Lung transplant outcome primary variable (Death all cause or Graft loss/death) are listed on the left column. The bold text refers to covariables that retained independent significant p-values in multivariate cox regression models*.

## Discussion

FcγR constitute a major marker of immune activation in response to infections, antibodies and CRP inflammatory ligands. Recent studies suggest that the engagement of FcR by the Fc fragment of alloantibodies can influence the cytotoxic activation level of NK cells toward the heart or kidney allograft. This study aimed to investigate whether the combined evaluation of *FCGR3A* [158F/V] and *FCGR2A* [131H/R] SNPs and biomarkers that index the level of CD16-dependent ADCC of NK cells might also be relevant to stratify patients at higher risk of lung allograft failure.

Our results show evidence that the *FCGR3A* [158V/V] genotype can be identified as a genetic marker that predisposes LTRs to acute rejection, independently of their DSA immunization status. Fifteen out of thirty patients with the *FCGR3A* [158V/V] genotype indeed developed acute rejection during the first trimester. Since this profile has been associated with higher NK cell responsiveness, we further investigated how it may relate to the NK cell mediated cytotoxic effects of DSA toward the lung allograft. Various studies have shown that the detection of circulating DSA with an ability to ligate the C1q component and activate the complement cascade can be associated with a greater risk of acute rejection and allograft loss after heart or kidney transplant. These complement-dependent mechanisms of DSA-mediated cytotoxic effects have also been recently documented in the lung transplant setting (19, 20). As previously reported (29), all C1q DSA detected at M3 were directed toward donor HLA-DQ alloantigens and C1q positive were shown to be significantly associated with acute rejection in the study cohort. A limitation of our study is the lack of elements allowing the distinction of cellular acute rejection (ACR) from ABMR. Endothelial deposition of C4d and microvascular inflammation were shown to be reliable markers of ABMR in renal and cardiac allografts, but the clinical relevance of C4d staining within lung allograft biopsies still remains controversial for lungs. These diagnostic criteria for ABMR were not always available on a routine basis at the time of evaluation until the Banff nomenclature released more accurate histologic features that characterize lung allograft grafts biopsies of DSA-positive LTRs.

However, the association between the *FCGR3A* [158V/V] genotype and presence of circulating DSA at M1 or at M3 did not appear to confer a higher risk of acute rejection, whatever their MFI and their C1q binding activity status, thus suggesting that the graft damage associated with the *FCGR3A* [158V/V] genotype is independent from DSA immunization status. This finding was unexpected, as we have previously shown that the intensity of antibody-dependent CD16 activation of NK cells, indexed by the non-invasive NK-CHAT, is significantly enhanced in recipients that bear the *FCGR3A* [158V/V] genotype and constitute a relevant cytotoxic mechanism sustaining toxic effects of DSA and allograft vasculopathy (48, 50). In contrast, NK-CHAT evaluation of DSA from LTRs actually shows evidence that the engagement of CD16 by LTx DSA is low when analyzed in reference to anti-HLA class II DSA that are found in the serum of kidney transplant recipients at time of ABMR diagnosis and that exhibit comparable HLA-DQ7 specificities and MFI values. This low potential of LTx DSA to induce CD16-mediated NK cell activation appears to be independent of their C1q binding activity and could not be associated with adverse outcome of LTx in the present study. As CD16 exhibits higher affinity for IgG1 and IgG3 alloantibodies, this failure of DSA to engage CD16 mediated cytotoxic functions may be due to the IgG2 and/or IgG4 isotypes of DSA, which have been shown to exhibit lower affinity for CD16. This finding may also indicate that the glycosylation pattern of IgG1 DSA interferes with the FcγRs-mediated recognition of the Fc fragment of IgG by immune cells or complement factors. These hypotheses are supported by the observation that only 30% of circulating DSA detected at M1 and at M3 were found to bind C1q in this study cohort and by previous findings that report high IgG4 levels in patients with cystic fibrosis lung disease (59). These data suggest that, in contrast to previous findings, the *FCGR3A* [158V/V] susceptibility genotype does not appear as a major mechanism of DSA-mediated lung allograft injury. CD16-dependent activation of NK cells in *FCGR3A* [158V/V] individuals that develop acute rejection may nevertheless be mediated by allo- or auto-antibodies that target non-HLA antigens and may not be revealed in the standardized NK-CHAT assay revealing CD16 cellular activation toward B lymphocyte cell targets. Several reports have indeed described ABMR lesions of the graft that occur in the absence of detectable levels of circulating DSA in the serum of kidney or heart transplant recipients. Antibodies directed against endothelial antigens or stress-induced antigens, such as vimentin, collagen V, Kα1 tubulin, AT1R, and MICA have also been reported in transplant recipients but the role of these non-HLA antibodies in the destruction and accelerated dysfunction of lung allograft remains poorly addressed (60). These reports have nevertheless raised interest in chronic injury resulting from humoral responses targeting non-HLA antigens, as these may be underestimated by the standard monitoring of patients' immunization status which is mainly restricted to the detection of anti-HLA alloantibodies (52). While the rate of acute rejection during the first year was previously identified as a risk factor for CLAD occurrence, occurrence of acute rejection in the first trimester could not be associated with an enhanced risk of developing chronic rejection nor with patient or graft survival in the present cohort. The F allelic variant of CD16 with low affinity for the Fc fragment of IgG was associated with the early development during the first month post-LTx of DSA that persist at M3. Persistence of DSA at M3 was thus less frequent in *FCGR3A* [158V/V] LTRs and was shown to be associated with lower survival times in LTR.

Our observations also identify a link between the presence of circulating DSA at M3 and the *FCGR2A* [131R/R] genotype, thus suggesting that FCGR2 polymorphisms may actually be associated with the persistence of harmful DSA at M3 rather than in the development of anti-HLA antibodies *per se*. The *FCGR2A* [131R/R] genotype was reported to be associated with shorter allograft survival in immunized kidney transplant recipients (KTR) (61, 62). We find that, independently of the risk associated with the *FCGR2A* [131R/R] genotype, detection and persistence of DSA at M3 constitute independent predictors of the adverse clinical composite outcome comprising CLAD or the patient's death. The persistence of DSA has been reported as a risk factor linked to BOS and to LTR death (16). A recent report showed that a majority of patients who were positive for *de novo* DSA during the first year after LTx developed BOS and were at higher risk of graft failure or death (63). In line with these reports, our study suggests that, independently of the presence of the susceptible *FCGR2A* [131R/R] genotype, the presence at M1 of DSA with high affinity for donor antigens (MFI DSA M1 > 18,750) can be identified as a risk factor associated with lower survival of LTRs.

The role of the complement-dependent pathogenicity of DSA is less well-documented in the lung transplant setting (19, 20). In a murine model of BOS, complement activation by antibodies to HLA class I was not required for the development of obliterative airway disease (OAD) that is similar to BOS in human LTx. Interestingly, in this study, at 90 days after LTx, only one out of 5 BOS patients had C1q DSA detected by SAFB whereas 3 out of 11 stable patients had C1q DSA. However, in our study, C1q DSA detected at M1 and M3 were mostly directed against HLA class II antigenic targets (90%) and do not appear as major contributors associated with the development of CLAD.

Although this is a limitation of the study, we cannot exclude the idea that auto-antibodies that target non-HLA antigens which were associated with the development of DSA, such as autoantibodies against K-α1 tubulin (K-α1T) and collagen V (ColV) (64), can participate in the chronic FcR-dependent reaction of recipient cells toward the lung allograft. This is supported by studies that report that development of col (V)-specific TH-17 cells may contribute to the pathogenesis of BOS (25, 59). Indeed, our standardized NK-CHAT evaluation of the DSA induced NK-cell activation was conducted toward B cell lines that express cognate HLA alloantigens. As B cells may not be relevant to evaluate the deleterious impact of non-HLA alloantibodies toward the lung allograft, this is a limit that prompts further studies that uses serum-coated lung epithelial or endothelial cells targets to evaluate NK cell ADCC.

*FCGR2A* [131R/H] is considered to be a heritable risk factor for a variety of infectious and inflammatory autoimmune diseases, including systemic lupus erythematosus, rheumatoid arthritis, malaria, multiple sclerosis, and anti-neutrophil cytoplasmic auto-antibody positive systemic vasculitis (65). The CD32 membrane receptor is expressed by a variety of immune cells that orchestrate the humoral immune response to pathogens, including B-lymphocytes, natural killer cells, macrophages, mast cells, and neutrophils. Its capacity to recognize IgGs bound to pathogens or infected cells has a protective effect against infections. While the *FCGR2A* allelic isoforms exhibit similar affinity for IgG1, the *FCGR2A* [131H/H] is the only FcγR variant that recognizes IgG2 subclasses, thus suggesting that the capacity to sense IgG2 antibodies may lead to impairment of pathogen surveillance in patients that lack the H allele. This SNP has been shown to play a role in the susceptibility to bacterial infections as *FCGR2A* [131H/H] individuals have greater potential to mediate IgG2-dependent bacterial phagocytosis than patients genotyped as *FCGR2A* [131R/R]. A distinct SNP (rs12746613) within the *FCGR2A* gene was previously associated with a higher risk of respiratory infections and mortality after LTx, but this variant was not associated with the risk of developing CLAD (34). Unlike other studies, our analysis of the present LTR cohort did not reveal any significant association between *FCGR2A* [131R/H] and occurrence of respiratory infections or the number of infection-related deaths. This discrepancy may relate to preventive and curative treatment of bacterial infections that differ between the transplantation centers. It may also reflect a complex interaction of these FCGR genotypes that encourages the overall survival of LTR for patients that have better capacities to thwart infections and overcome early acute rejection events. The observed finding of a inverse link between the *FCGR2A* [131R/R] susceptibility genotype and presence of the FCGR3A-V allele encoding the CD16 receptor variant with higher affinity for the IgG Fc fragment in LTRs, may in part explain a lack of association of the FcCR3A-VV genotype with DSA- mediated chronic lung allograft dysfunction. In this study, the presence of the *FCGR2A* [131H/H] was observed to be strongly associated with the presence of the “high IgG1 responder” *FCGR3A* [158V/V] genotype, and such linkage disequilibrium may in part explain how this intricate distribution of susceptible and protective FCR SNPs may participate in the complex tuning of the host immune response to early infectious and humoral challenges and may be associated with enhanced survival and lower rates of CLAD in patients who have the protective *FCGR2A* [131H/R or H/H] genotype, notably in female LTRs.

In addition to this protective role against pathogens, the SNP dependent affinity of CD32 for the Fc fragment of IgG and/or CRP ligands was also identified as promoting inflammation. CD32-dependent triggering of immune cells is in part conditioned by the polymorphism and the expression profile of this functional receptor at the surface of immune cells. As is expressed by most leukocytes/macrophages that infiltrate the lung graft, *FCGR2A* [131R/H] could also influence the acquisition of an inflammatory-activated profile that favors tissue recruitment of activated lymphocytes to the lung (66). Interestingly, the *FCGR2A* [131R/R] susceptible genotype identified in this study has been associated with higher CRP binding avidity for the CD32 receptor expressed at the surface of monocytes and neutrophils.

Considering the growing evidence of the key role of CRP as an inflammatory mediator involved in the development of atherosclerosis and endothelial dysfunction, it is expected that CRP may be more powerful in triggering the pro-inflammatory function of CD32-expressing cell subsets such as platelets, endothelial cells, monocytes, and leukocytes in *FCGR2A* [131R/R] LTRs.

In conclusion, these data highlight that *FCGR2A* and *FCGR3A* polymorphisms constitute predisposing factors that are associated with the outcome of lung allografts. This study suggests that the combined assessment of the FCGR genotype and CRP or IgG ligands is thus an intriguing prospect to further decipher the complex mechanisms that shape the alloimmune and inflammatory responses in response to infectious and humoral threats. As shown in other organ transplant settings, our study indicates FCGR genotyping may favor early stratification of patients at risk and may create new perspectives to adapt personalized preventive and therapeutic approaches to prevent adverse outcomes of lung transplants.

## Data Availability

The raw data supporting the conclusions of this manuscript will be made available by the authors, without undue reservation, to any qualified researcher.

## Ethics Statement

All patients from the French cohort (COLT, Cohort in Lung Transplantation, l'Institut du Thorax, INSERM UMR1087/CNRS UMR 6291, CNIL 911142) were recruited in this study and gave their written informed consent to participate to the study in accordance with the Declaration of Helsinki. A group of 184 healthy unrelated of volunteer French bone marrow donors were also recruited to constitute a control cohort allowing analysis of FcγR genotype. Blood donations were collected in the Etablissement Francais du Sang, in accordance with BSL-2 practices. A medical interview was carried out prior to blood donation to exclude donors with medical contraindications. This study was carried out in accordance with the French Public Health Code (art L1221-1), approved by institutional ethics committee and conducted in compliance with the Good Clinical Practice Guidelines, declaration of Helsinki and Istanbul.

## Author Contributions

CP and PPa designed and coordinated the study, analyzed the data, and wrote the paper. PPe, LL, and JD performed experiments. AL contributed to the methodological and statistical analysis. MP, AB, FD-G, and JC contributed to the research design. PT and MR-G contributed to the collection of patient material and to the clinical aspects of the study.

### Conflict of Interest Statement

The authors declare that the research was conducted in the absence of any commercial or financial relationships that could be construed as a potential conflict of interest.

## Abbreviations

HLA: Human Leukocyte Antigen
LTx: Lung Transplant
LTRx: Lung Transplant Recipient
Abs: Antibodies
Ag: Antigen
DSA: Donor-Specific Antibodies
BOS: Bronchiolitis Obliterans Syndrome
CLAD: Chronic Lung Allograft Dysfunction
ADCC: Antibody dependent cell cytotoxicity
CT: Computed Tomography
RAS: Restrictive Allograft Syndrome
ABMR: Antibody Mediated Rejection
ACR: Acute Cellular Rejection
SAFB: Single-Antigen Flow Beads
D: Days
M: Month
CMV: Cytomegalovirus
MFI: Mean Fluorescence Intensity
OS: Overall Survival
DFS: Disease Free Survival
CDC: Complement-Dependent Cytotoxicity
CF: Cystic Fibrosis.
